# Effect of amifostine, a radiation-protecting drug, on oxygen concentration in tissue measured by EPR oximetry and imaging

**DOI:** 10.3164/jcbn.15-130

**Published:** 2017-03-25

**Authors:** Megumi Ueno, Shingo Matsumoto, Atsuko Matsumoto, Sushma Manda, Ikuo Nakanishi, Ken-ichiro Matsumoto, James B. Mitchell, Murali C. Krishna, Kazunori Anzai

**Affiliations:** 1 Radio-Redox-Response Research Team, Advanced Particle Radiation Biology Research Program, Research Center for Charged Particle Therapy, National Institute of Radiological Sciences, 4-9-1 Anagawa, Inage-ku, Chiba-shi, Chiba 263-8555, Japan; 2 Radiation Biology Branch, Center for Cancer Research, National Cancer Institute, Building 10, NIH, Bethesda, MD 20892-1002, USA; 3 Nihon Pharmaceutical University, 10281 Komuro, Ina-machi, Kitaadachi-gun, Saitama 362-0806, Japan

**Keywords:** amifostine, hypoxia, radioprotector, EPR oximetry, EPR imaging

## Abstract

Effect of amifostine, a radiation-protecting drug, on muscle tissue partial pressure of oxygen was investigated by electron paramagnetic resonance spectroscopy and imaging. When amifostine was administered intraperitoneally or intravenously to mice, the linewidth of the electron paramagnetic resonance spectra of the lithium octa-*n*-butoxy-substituted naphthalocyanine implanted in the mouse leg muscle decreased. Electron paramagnetic resonance oximetry using a lithium octa-*n*-butoxy-substituted naphthalocyanine probe and electron paramagnetic resonance oxygen mapping using a triarylmethyl radical paramagnetic probe was useful to quantify pressure of oxygen in the tissues of living mice. The result of electron paramagnetic resonance oximetric imaging showed that administration of amifostine could decrease pressure of oxygen in the muscle and also tumor tissues. This finding suggests that lowering pressure of oxygen in tissues might contribute in part to the radioprotection of amifostine.

## Introduction

Several aminothiol compounds are known as radio-protectors, such as cysteine (H_2_NCH(CH_2_SH)COOH) and cysteamine (H_2_NCH_2_CH_2_SH).^(1,2)^ Amifostine (WR-2721: H_2_N(CH_2_)_3_NH(CH_2_)_2_SPO_3_H_2_), a powerful and clinically approved radiation-protecting drug, was developed initially by the Walter Reed Army Medical Center (Bethesda, MD). Amifostine is also a phosphorylated pro-drug, which can be activated *in vivo* to the -SH form (WR-1065: H_2_N(CH_2_)_3_NH(CH_2_)_2_SH). Administration of amifostine before irradiation has an efficient radioprotective effect.^(3–5)^ The mechanism(s) of amifostine radioprotection include scavenging of secondary radicals generated by radiation exposure, donation of an electron to carbon-centered radicals resulting from radiation exposure (chemical repair), stabilization of DNA repair, inhibition of cell-cycle progression, and hypoxia induction.^(6–8)^ Therefore, it is postulated that *in vivo* radioprotection by the amifostine might be due to the synergistic effect of those individual functions.

Using tritium-labeled misonidazole binding for hypoxia detection, Allalunis-Turner *et al.*^(9)^ reported induction of bone marrow hypoxia in mice following injection with amifostine. This finding suggested that amifostine-mediated radioprotection may in part be a result of hypoxia induction. However, it is not clear whether hypoxia induction occurs in other tissues/organs following amifostine treatment.

Tissue oxygen concentration is an important factor in the response of tissue to radiation exposure (i.e., a lower oxygen concentration reduces the radiation effect, which is the so-called oxygen effect). Oxygen concentration in tumor/cancer tissues greatly affects the outcome of radiotherapy. Many tumors have significant hypoxic fractions, and show radio-resistant behavior. In addition, the harmful effects of radiation on normal tissues are also related with the relatively higher (actually normal) oxygen concentration in normal tissue. Approximately 70% of the biological effect of radiation, such as X-ray, γ-ray and/or β-ray, is indirect, mediated by reactive oxygen species (ROS). The hydroxyl radical and hydrogen atom can be produced primarily by direct ionization and/or excitation of a water molecule. Hydrogen atoms and part of hydroxyl radicals can react with dissolved oxygen in tissues to make other ROS, i.e., superoxide, hydrogen peroxide, etc. Hence, the biological effect of radiation is greatly affected by the oxygen concentration in irradiated tissue.

Electron paramagnetic resonance (EPR) oximetry is a noninvasive and sensitive method to measure oxygen concentration using a stable free radical species as an oximetric molecular probe.^(10)^ EPR is a magnetic resonance modality to measure absorption of microwave/radio frequency by free radicals, i.e., unpaired electrons. The linewidth of EPR spectrum of the oximetric probe can be sensitively broadened according with oxygen concentration in a sample. Since oxygen (i.e., O_2_) has two stable unpaired electrons on the molecule, collision of free radicals with each other can cause shortening of the EPR relaxation time and broadening of the EPR linewidth. EPR oximetry can combined with the EPR spectral-spatial imaging technique, and then 3D oxygen mapping can be carried out in a sample and/or an animal.^(11)^

In the present study, we examined the effect of amifostine on the oxygen concentration in a mouse leg muscle by EPR oximetry using a particle-type stable radical, lithium octa-*n*-butoxy-substituted naphthalocyanine (LiNc-BuO),^(12)^ as a paramagnetic probe. EPR oximetry is a useful technique for non-invasive and quantitative measurement of tissue oxygen concentration.^(13)^ The EPR linewidth of paramagnetic probes can linearly respond to the oxygen concentration surrounding the probes. Since the combination of an imaging modality with EPR oximetry can provide quantitative oxygen mapping in living animals,^(11)^ we obtained oxygen mapping of a tumor-bearing mouse leg before and after amifostine treatment by time-domain EPR imaging using a triarylmethyl-type stable radical, tri[8-carboxy-2,2,6,6-tetrakis(2-hydroxymethyl)benzo[1,2-*d*:4,5-*d'*]bis(1,3)dithio-4-yl]methyl radical (known as Oxo63),^(14)^ as an oxygen probe.

## Materials and Methods

### Chemicals

LiNc-BuO was synthesized according to the literature.^(12)^ Amifostine was purchased from Yamanouchi Pharmaceutical Co., Ltd. (merged with Fujisawa Pharmaceutical Co., Ltd. in April 2005 as Astellas Pharma Inc., Tokyo, Japan). Carbogen (gas composition of 95% O_2_ and 5% CO_2_) was purchased from Takachiho Chemical Industrial Co., Ltd. (Tokyo, Japan). Oxo63 was obtained from GE Health Care (Milwaukee, WI).

### Animals

Healthy 7-week-old female C3H mice were supplied by Japan SLC, Inc. (Shizuoka, Japan) for EPR spectroscopy and by Frederick Cancer Research Center, Animal Production (Frederick, MD) for EPR imaging. Animals were housed five per cage in climate controlled (23 ± 1°C and 55 ± 5% humidity) circadian rhythm-adjusted (12-h light–dark cycle) rooms and were allowed food and water *ad libitum*. EPR spectroscopic experiments were carried out in compliance with and approved by the Animal Use Committee of the National Institute of Radiological Sciences, Chiba, Japan. LiNc-BuO was implanted when the mice were 10 weeks old. Body weight of mice measured immediately prior to the experiments was in the range of 23–27 g. EPR imaging experiments at the National Cancer Institute were carried out in compliance with the Guide for the Care and Use of Laboratory Animal Resources, National Research Council, and approved by the National Cancer Institute Animal Care and Use Committee. Mice were used for experiments at 10–12 weeks old. Their body weight before the experiments was in the range of 26–30 g.

### Standard pO_2_ calibration curve

The calibration curve for LiNc-BuO oximetry was obtained according to the previous report with some modifications.^(13)^ An aliquot of LiNc-BuO crystals suspended in PBS was placed in a gas-permeable Teflon tube, which was placed on a single loop surface coil resonator (5.5 mm i.d.) with the crystals at the center of the coil. The resonator with the sample was covered with a plastic tube and warmed up to 36–37°C using a combination of hot air and an IR lamp. The EPR signals of the LiNc-BuO crystal were measured by l-band (1.1 GHz) CW EPR (JEOL, Tokyo, Japan) under several gas flow conditions, including 21% (medical air), 10%, (O_2_/N_2_ percentage composition), and 0% (N_2_). The measurements were performed after at least 30 min equilibration under each gas flow condition at the rate of 1 L/min. EPR data acquisition was controlled by the WIN-RAD ESR Data Analyzer System (Radical Research, Inc., Hino, Japan), which is composed of an A/D converter, a PC, and software to interpret EPR data. The experimental settings were as follows: microwave frequency = 1.1 GHz, scan rate = 0.75 mT/min, time constant = 0.03 s, and field modulation frequency = 100 kHz. The microwave power (0.25–1.0 mW) and field modulation width (0.0125–0.05 mT) were adjusted for each measurement to avoid saturation and artificial line broadening. The acquired EPR spectrum was analyzed using an in-house line-fitting program, and the Lorenzian line shape was fitted. The linewidth of the fitted Lorenzian line was determined. A calibration curve for LiNc-BuO oximetry was obtained by plotting the linewidth against the oxygen concentration. A calibration curve was obtained for each batch of LiNc-BuO crystals.

### Implantation of LiNc-BuO crystal

Each mouse was anesthetized by administering 1.5% isoflurane in medical air (flow rate of 1 L/min) through a nose cone. The fur in the femoral region was removed by shaving. To ensure implantation of LiNc-BuO, an excessive amount (5–10 mg) of LiNc-BuO crystals was placed in the tip of a 20-gauge injection needle. The needle was injected into the femoral region of the animal, and the LiNc-BuO crystals were pushed out using a smooth-fitting piston. LiNc-BuO was implanted in both the right and left femoral muscles of the mouse.

### pO_2_ measurement in mouse muscle

A mouse was anesthetized with 1.5% isoflurane in medical air flow (1 L/min) and placed in a special mouse holder. The mouse legs and lower abdomen were fixed by adhesive tape to the holder. The 1.1 GHz single loop surface coil (5.5 mm i.d.) was placed in the region where the LiNc-BuO crystal was implanted. The EPR conditions were the same as above except that the micowave power was 0.25 mW and field modulation width was in range of 0.016–0.02 mT. The core body temperature in the rectum was monitored by a nonmagnetic probe (FISO Technologies Inc., Quebec, Canada) during EPR measurements. The core body temperature of the experimental animal was regulated at 37 ± 1°C using hot air and an IR lamp during EPR experiments. Carbogen was applied throughout respiration. Amifostine was applied through i.p. or i.v. injection through a line cannulated into the tail vein or inserted into the peritoneal cavity beforehand.

### Oxygen mapping by time-domain EPR imaging technique

EPR imaging was performed using the 300 MHz time-domain EPR imager (Radiation Biology Branch, National Cancer Institute, National Institutes of Health, Bethesda, MD). Mouse squamous cell carcinoma SCCVII tumor was formed by injecting 2 × 10^5^ cells subcutaneously into the right hind legs of CH3 mice 10 days before the experiments. The tumor size on the day of the experiment was ≈1.2 cm × 1.2 cm × 1.2 cm. The tumor-bearing mouse was anesthetized with 1.5% isoflurane in medical air flow (1 L/min) and both legs were fixed by adhesive tape to the special mouse holder. According to the previous report,^(11,15)^ a water-soluble paramagnetic oxygen probe, Oxo63, was given as a 1.125 mmol/kg bolus followed by 0.04 mmol/kg/min continuous infusion, which was started just before commencing image data acquisition. Four-dimensional (4D, i.e., 1D spectral + 3D spatial) time-domain EPR spectral-spatial imaging (SSI)^(11)^ was performed repeatedly. Scan time for one 4D spectral-spatial image was around 10 min. The 4D spectral-spatial data set was assembled with 3 maximum gradient sets (1.4, 1.14, and 0.96 Gauss/cm). Each maximum gradient set was assembled with 21 × 21 × 21 = 9261 gradient steps. After the acquisition of first 4D data set, 1.5 mmol/kg amifostine was administered by intravenous injection. Three-dimensional (3D) oxygen mapping was recalculated from 4D SSI data.

## Results and Discussion

**Fig. 1:**
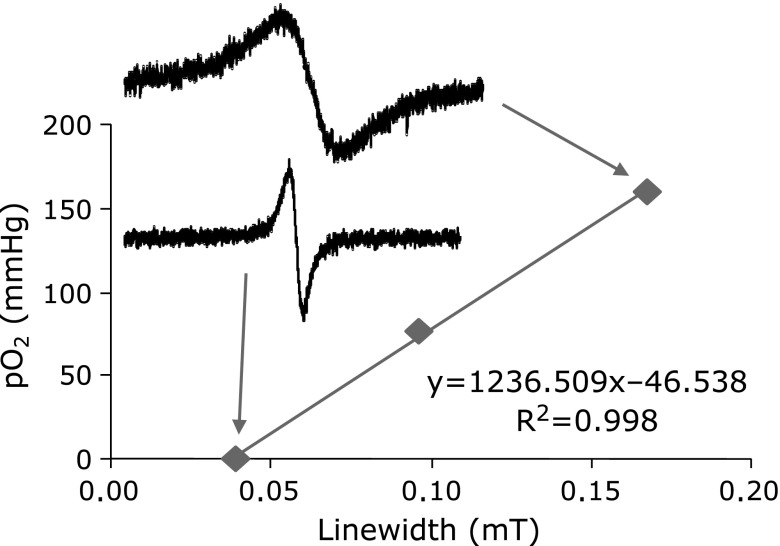
Calibration curve of LiNc-BuO oximetry. EPR linewidth of LiNc-BuO versus pO_2_ under various gas conditions, including 21% (room air), 10%, and 0% oxygen was plotted. EPR signals were measured using an l-band EPR spectrometer equipped with a 1.1 GHz surface coil resonator. EPR conditions were as follows: microwave frequency, 1.1 GHz; scan rate, 0.05–0.25 mT/min; time constant, 0.03 s; field modulation frequency, 100 kHz; microwave power, 0.5–4 mW; field modulation width, 0.0125–0.05 mT. The spectra shown in the figure were measured under 0 and 21% gas conditions. The calibration curve need to be measured for each lot of LiNc-BuO crystals, since the intrinsic EPR linewidth of the crystal is slightly variable by the lot of the crystal.

First, a standard calibration curve for *in vivo* EPR oximetry was prepared. Considering the physiological condition, i.e., gas solubility in physiological fluid at 37°C, LiNc-BuO crystals suspended in PBS were measured in the range of 36–37°C. The EPR linewidth of LiNc-BuO was broadened by increasing the oxygen concentration of flowing gas, and responded linearly (Fig. 1). This plot was used as the standard pO_2_ calibration curve for the same preparation of LiNc-BuO in the experiments described below.

**Fig. 2:**
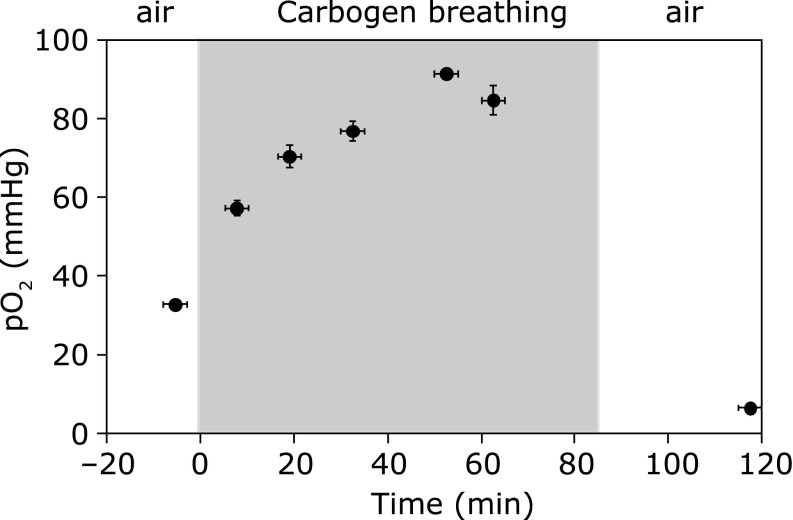
Effect of carbogen breathing on pO_2_ in leg muscle of a mouse. Carbogen breathing started from time 0, and ended at 85 min. The gas flow rate was 1 L/min. Values are the average ± SD of sequential 3 measurements.

In the next step, the response of implanted LiNc-BuO in muscle tissue of the hind leg of the mouse was tested. Carbogen breathing by mice broadened the linewidth of LiNc-BuO in the muscle of the mouse leg, and the linewidth of LiNc-BuO narrowed again when the breathing gas was switching back to air (Fig. 2). The pO_2_ values were obtained from the linewidth of LiNc-BuO using the pO_2_ calibration curve. The pO_2_ level in muscle increased gradually when the mouse was breathing carbogen, and then decreased when the breathing gas was switched back to air. The result is similar to the experiment, which used lithium phthalocyanine (LiPc) as the oxygen probe,^(13)^ except in one point that the pO_2_ level at 120 min, 35 min after switching the gas back to air from carbogen, was markedly lower than the pO_2_ level before breathing carbogen in this experiment. In the previous experiment using LiPc, pO_2_ in muscle tissue after switching gas back to air from carbogen decreased to the same or a slightly higher level than before breathing carbogen. This may be due to the differential distribution of different types of oxygen probes, i.e., LiPc and LiNc-BuO. LiPc, which cannot enter cells, is only an extracellular oxygen probe. LiNc-BuO micro-crystals are, however, much inert in biological systems, and then sub-micron or smaller sized particles of LiNc-BuO can be taken into cells.^(12,16,17)^ Therefore LiNc-BuO particles implanted in the mouse tissue may distribute to extra- and partly intracellular spaces, and then the EPR linewidth of the LiNc-BuO in the mouse tissue may be able to reflect extra- and partly intracellular oxygen environments. The intracellular oxygen concentration once put up by breathing carbogen may be pull down rapidly due to intracellular oxygen consumption when the breathing gas was switched back to air; however, extracellular oxygen concentration may be stagnated because no or less oxygen consumption factors in the extracellular space. The EPR oximetry is sensitive for lower oxygen concentration due to its principle. An overlapping narrower EPR line, which is reflecting intracellular pO_2_, on a broader line, which is reflecting extracellular pO_2_, can make pO_2_ underestimated. In addition, normal muscle tissue pO_2_ may be variably sensitive by the condition of the anesthesia and/or the fixing of the animal. The possible mechanisms and also the accuracy of this observation are still under investigation.

**Fig. 3:**
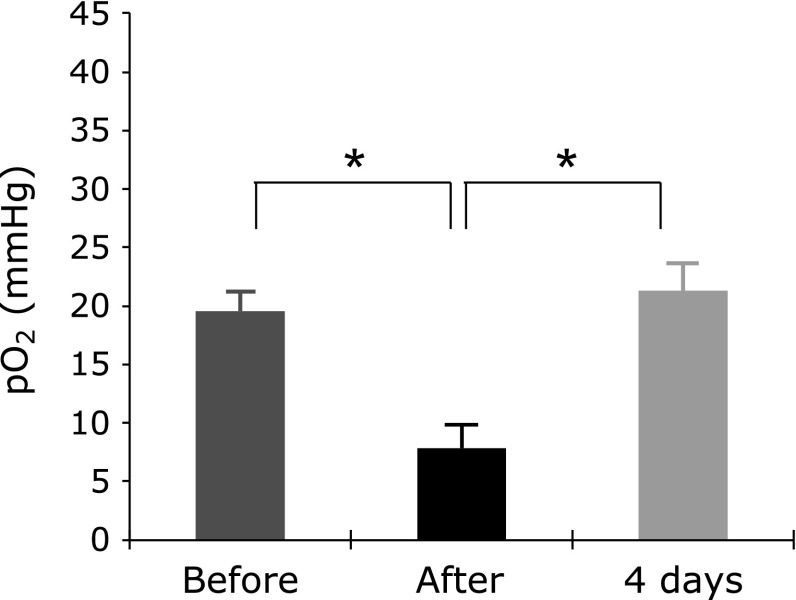
Effect of intraperitoneal administration of amifostine on pO_2_ in the muscle of mouse hind leg. EPR was measured before, 60 min after, and 4 days after administration of amifostine. Values are the average ± SD of 5 mice. * indicates significant difference at *p*<0.001.

**Fig. 4:**
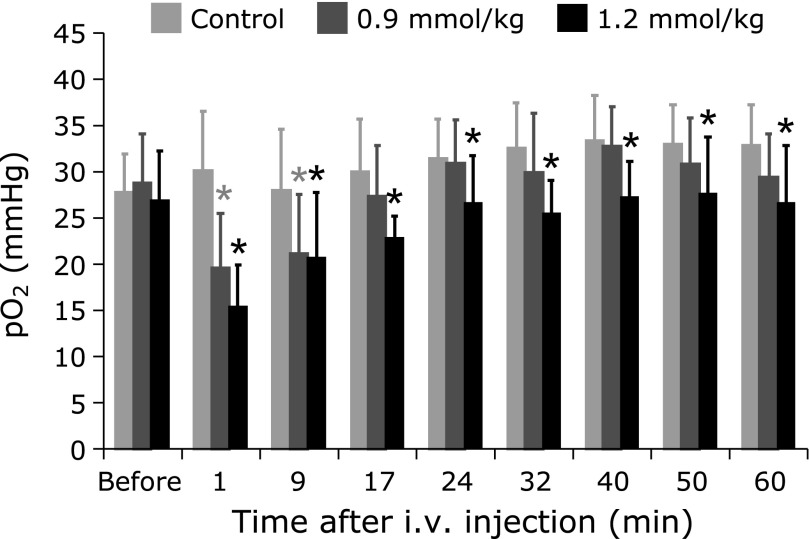
Effect of intravenous administration of amifostine on pO_2_ in muscles of mouse hind leg. EPR was measured before administration and then repeatedly until 60 min after administration. The control group was administered 0.9% NaCl saline instead of amifostine solution. Values are the average ± SD of 5 mice. * indicate significant difference between the administered and control groups at *p*<0.05.

Intraperitoneal administration of amifostine gradually decreased the linewidth of the EPR spectra of LiNc-BuO in the mouse leg, indicating that amifostine decreased pO_2_ in the leg muscle (Fig. 3). The decrease of pO_2_ lasted more than 2 h without a significant change in the core body temperature (data not shown). The decrease of pO_2_ induced by intraperitoneal administration of amifostine recovered within 4 days (Fig. 3). Intravenous administration of amifostine also decreased pO_2_ in the muscle in a dose-dependent manner (Fig. 4). The onset and recovery of the pO_2_ changes were faster than those observed when amifostine was administered intraperitoneally.

The plasma half-life of intravenously injected amifostine has been reported at several minutes following injection.^(18)^ Amifostine can be distributed in muscle at a similar concentration level as in blood.^(19)^ It has been also reported that normal tissues actively concentrate amifostine.^(20)^ Those facts suggest that the distribution of amifostine from blood to whole body normal tissues is relatively fast. In that case, distribution of amifostine in the opposite direction, i.e., from tissue to blood, may be slow. Such kinetic characteristics of amifostine may make the sustained hypoxia by the intraperitoneal administration and the brief hypoxia observed by the intravenous administration.

Although Gray has regarded oxygen removal by thiol compounds,^(21)^ disrupting the oxygen supplies may make a huge contribution to relatively quick hypoxia formation by the intravenous administration of amifostine. In the present study, a brief temporal decrease in the body temperature of the mouse (data not shown) was observed when the amifostine was injected intravenously, even though the mouse body temperature was controlled during the experiment. Such decrease in body temperature may be suggestive of amifostine’s role to weaken the peripheral blood flow.

**Fig. 5:**
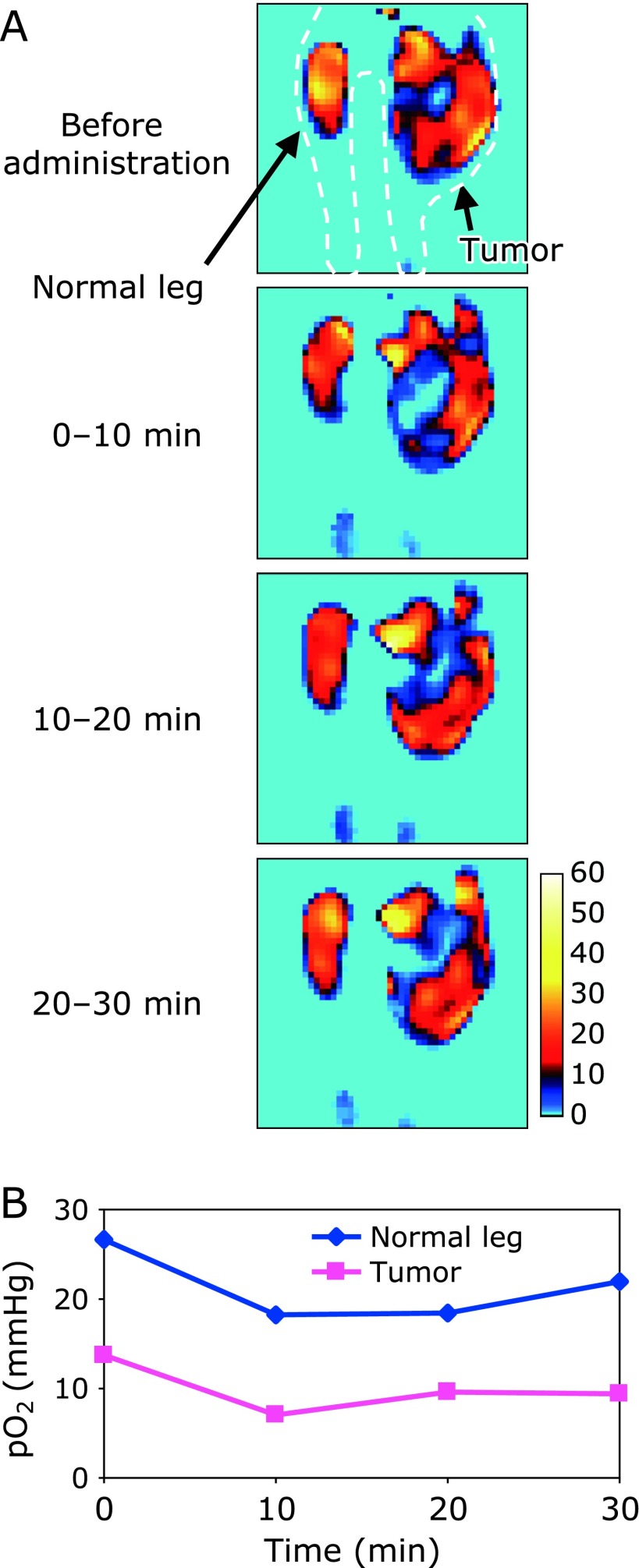
Oxygen mapping in tumor-bearing mouse legs before and after administration of amifostine. (A) Sequential pO_2_ mapping of mouse legs. (B) Time course of average pO_2_ value in normal and tumor tissue.

Fig. 5 shows the result of EPR oxygen mapping. 4D SSI data were obtained every 10 min and 3D oxygen mapping was reconstructed from each 4D SSI data set. A slice including both the normal and tumor bearing legs of the mouse was extracted from each 3D oxygen map. The four slices shown in Fig. 5 were extracted from exactly the same position of each data matrix. The hypoxic region in the tumor obviously increased after administration of amifostine, and then gradually shrunk over time. Similarly, average pO_2_ values in both tissues decreased after administration of amifostine, and then gradually recovered. Although several previous papers reported the normal tissue-specific radioprotection effect of amifostine,^(22–24)^ pO_2_ was reduced both in normal and tumor tissues. Active concentrating of amifostine into normal tissues,^(20)^ rather than pO_2_ differences between normal and tumor tissues, may convincingly explain the mechanism of the normal tissue specificity of radioprotective effects.

Superoxide may be an effective attacker during radiation damage since a superoxide dismutase mimic, TEMPOL, can inhibit oxygen-dependent radiation-induced damage,^(25)^ although it has been reported that the primary product of ionizing radiation in a living body is hydroxyl radical.^(26)^ Generally, -SH compounds are known as superoxide scavengers. It has been reported that glutathione can react with superoxide with a rate constant of 200 M^−1^s^−1^.^(27)^ Amifostine has a greater radio-protective effect than glutathione, which is a natural thiol antioxidant.^(28)^ Even though other factors rather than a free radical scavenging effect may strengthen the radioprotective effect of amifostine, part of the radioprotective effect of amifostine may be due to its radical scavenging effect of the activated -SH form during radiation.

It has been reported that i.p. treatment of amifostine 30 min before irradiation decreased EPR signal decay rate of carbamoyl-PROXYL in upper abdomen of X-irradiated mice.^(29)^ Elas *et al.*^(30)^ also reported that treatment via i.p. or oral route with amifostine prior to radiation slowed the EPR signal decay rate of carboxy-PROXYL, which is a membrane impermeable molecule, in mice by 23% or 18%, respectively. Those results suggest that the *in vivo* clearance of nitroxyl radical probes were retarded by amifostine and give an expectation of a reduction in tissue blood flow. It has been reported that side-effects associated with amifostine are nausea, vomiting and hypotension.^(31)^ The main potentially dose-limiting adverse effect is hypotension, which is often asymptomatic.^(32)^ Further studies showed that hypotension after administration of amifostine is mediated by WR-1065, which is a metabolite of amifostine, and appears to result from direct relaxation of vascular smooth muscle, but not related to nitric oxide.^(33)^ Several cases of anaphylactoid reactions to amifostine have been reported.^(34–36)^ Systemic hypotension may cause insufficiency in the peripheral circulation and tissue hypoxia, while unequivocal mechanism of amifostine induced tissue hypoxia is still not known.

When amifostine was administered intraperitoneally or intravenously to the mice, the linewidth of the EPR spectra of LiNc-BuO, a paramagnetic oxygen probe, implanted in the mouse leg muscle, decreased. In the present study, we also clearly showed directly pO_2_ reduction in normal and tumor tissues by EPR oximetric imaging. Since the radiosensitivity of cells depends on the oxygen concentration and hypoxic cells are radio-resistant, the results of this study suggest that the lowering of pO_2_ in normal muscle tissues after administration of amifostine can contribute in part to the radioprotection effect of amifostine.

## Conclusion

EPR oximetry using a LiNc-BuO probe and EPR oxygen mapping using Oxo63 was useful to quantify pO_2_ in the tissues of living mice. Administration of amifostine significantly decreased pO_2_ in the muscle transiently. This finding suggests that changing the oxygen localization and induction of hypoxia can be an important factor for the radioprotective effect of amifostine.
